# Electronic Patient‐Reported Health‐Related Quality of Life Trajectories in ICU Survivors: A Multicentre Pilot Study‐Within‐a‐Trial Protocol

**DOI:** 10.1111/aas.70342

**Published:** 2026-09-29

**Authors:** Elisa Zoe Battistelli, Benjamin Skov Kaas‐Hansen, Anders Granholm, Ronni Thermann Reitz Plovsing, Bodil Steen Rasmussen, Anders Perner, Maj‐Brit Nørregaard Kjær

**Affiliations:** ^1^ Department of Intensive Care Copenhagen University Hospital—Rigshospitalet Copenhagen Denmark; ^2^ Università degli Studi Milano‐Bicocca Milano Italy; ^3^ Section of Biostatistics, Department of Public Health University of Copenhagen Copenhagen Denmark; ^4^ Department of Anaesthesiology and Intensive Care Copenhagen University Hospital—Amager and Hvidovre Hvidovre Denmark; ^5^ Department of Clinical Medicine University of Copenhagen Copenhagen Denmark; ^6^ Department of Anaesthesiology and Intensive Care Aalborg University Hospital, Aalborg University Aalborg Denmark; ^7^ Department of Clinical Medicine Aalborg University Aalborg Denmark

## Abstract

**Background:**

Patient‐important outcomes beyond mortality, particularly health‐related quality of life (HRQoL), are frequently used in intensive care unit (ICU) trials. However, HRQoL assessment remains challenging due to methodological complexities, including poorly defined recovery trajectories, lack of consensus on measurement instruments, complexity of statistical analysis, and missing data. Electronic patient‐reported outcomes (ePROs) may improve data collection and efficiency, though their feasibility in ICU survivors is uncertain.

**Methods:**

This protocol outlines a longitudinal, multicentre pilot study‐within‐a‐trial aimed at evaluating the feasibility of ePRO‐based HRQoL follow‐up and exploring a trajectory‐based approach to HRQoL characterisation after critical illness. We plan to enrol 100 participants already undergoing 180‐day HRQoL follow‐up in the Intensive Care Platform Trial (INCEPT) to complete monthly EQ‐5D‐5L surveys delivered by text messages from 6 to 12 months after randomisation. Feasibility outcomes include enrolment and response rate, time to completion, reminder‐dependency, attrition pattern, agreement between modes of collection, and accessibility. Agreement between telephone‐ and ePRO‐based assessments at 180 days will be evaluated at the group and at the individual level. Longitudinal EQ‐5D‐5L index values and visual analogue scale trajectories will be analysed using area‐under‐the‐curve methods based on linear interpolation. Scenario‐based sensitivity analyses will assess the potential impact of unobserved mortality among dropouts.

**Discussion:**

We hypothesise that ePRO‐based repeated assessments of HRQoL will support the goal of optimised data collection methods, while ensuring resources control. Moreover, identification of a candidate approach to HRQoL characterisation may shed light into the full recovery trajectory of ICU survivors and improve the interpretation and clinical relevance of HRQoL outcome assessments.

## 1 Introduction

### 1.1 Background

Patient‐important outcomes other than mortality are increasingly recognised as central endpoints in clinical trials in adult intensive care patients, reflecting a focus on patient‐centered outcomes, survivorship, and a deeper understanding of long‐term sequelae of critical illness [1]. These outcomes are now incorporated into clinical trial guidelines [2, 3], and are gaining a central role in core outcome sets developed for different intensive care unit (ICU) populations [4, 5, 6]. Health‐related quality of life (HRQoL) is one of the most widely used patient‐important outcomes. It is multidimensional and, typically, self‐reported, a so‐called patient‐reported outcome (PRO), so better suited for characterising complex physical and emotional states than simpler outcome measures such as survival. Despite the improved clinical relevance, HRQoL remains suboptimally utilised in intensive care research [7], likely because designing trials around these outcomes introduces methodological complexities.

Indeed, HRQoL has some operationalisation caveats. Its trajectory after ICU discharge is yet to be clarified, as some longitudinal studies suggest convergence towards HRQoL of the general population [8]. Others report persistent reduced HRQoL values 5 years after critical illness [9], indicative of accelerated functional decline or relapsing‐recurring episodes [10]. Moreover, a paradoxical negative effect of certain survival‐improving interventions on HRQoL has been observed [11], highlighting the complex nature of this PRO [12]. Measurement of HRQoL is further complicated by the lack of consensus on a patient‐reported outcome measure (PROM). Several instruments exist, including the EQ‐5D 5 level (EQ‐5D‐5L) developed by the EuroQol group [13, 14], which is one of the most widely used [7]. It comprises five domains, each with five levels together with the visual analogue scale (EQ VAS) ranging from 0 to 100. Responses can be summarised in a preference‐weighted index value. Beyond operationalisation, challenges extend to statistical analysis of HRQoL. There is limited guidance on the most appropriate methods for EQ‐5D statistical analysis of treatment effects [15], creating great heterogeneity in practice [16]. In addition, key distributional features of HRQoL data [15, 17], such as skewness, ceiling effect, and discreteness, are often overlooked when selecting the analytical models, potentially leading to violation of assumptions for statistical analyses. All these aspects reduce comparability of results. Moreover, missing HRQoL data due to loss‐to‐follow‐up is frequent and poses a well‐known challenge in randomised clinical trials (RCTs) [7, 18]. Handling of missingness remains inconsistent [19], and chosen methods very often go unreported or unjustified [18]. Given that HRQoL data are unlikely to be missing completely at random [20], inadequate handling may introduce bias and compromise validity. Similarly, truncation due to death poses a relevant methodological challenge in RCTs conducted in high mortality populations, especially when mortality differs between groups, thus introducing bias if not handled [21].

In this context, methods to optimise data collection and retention are warranted. Electronic patient‐reported outcomes (ePROs) refer to PRO measures reported by patients through electronic platforms and they are increasingly integrated into clinical trials [22]. They can facilitate data completeness [23], enable real‐time monitoring and traceability of data entry and integrity [24], and reduce both participant and personnel burden [25]. Available evidence from two meta‐analyses comparing electronic and paper forms supports comparability across different modes of collection [26, 27], when best practices for implementation and instrument migration are adhered to [28]. However, ePRO raises the issue of accessibility [29], ongoing training [30], and their adoption in clinical trials is still limited [22]. This is especially true for the adult ICU population, as evidence of ePRO‐based follow‐up in this context is lacking.

### 1.2 Objectives

We will conduct an observational, prospective pilot study‐within‐a‐trial in a mixed ICU population using monthly web‐based ePRO HRQoL assessments delivered via text message (short message service, SMS) over a 12‐month follow‐up period to evaluate the feasibility of long‐term repeated measurements of HRQoL and explore the EQ‐5D‐5L and EQ VAS area‐under‐the‐curve (AUC) data as candidate summary outcome measures that may better characterise and capture changes over time, similarly to quality‐adjusted life years (QALYs) analysis in health economic evaluations.

## 2 Methods

This protocol is written in accordance with the Strengthening the Reporting of Observational Studies in Epidemiology (STROBE [31]) statement and the CONSORT statement: extension to randomised pilot and feasibility trials [32]. Completed checklists are included in the supplements (Tables S1 and S2).

### 2.1 Study Design and Setting

This pilot study will be an observational, prospective cohort study conducted at three mixed ICUs in Denmark, at Copenhagen University Hospital—Rigshospitalet (coordinating site), at Aalborg University Hospital, and at Copenhagen University Hospital—Hvidovre Hospital. It is a study‐within‐a‐trial (SWAT [33]), embedded in the Intensive Care Platform Trial (INCEPT, EUCT 2024–516,208–41‐00) [34], a domain‐based international platform trial focused on commonly used interventions in adults acutely admitted to the ICU. This study focuses on HRQoL, which is one out of six core platform‐level outcomes [4]. INCEPT patients are followed daily while in‐hospital for up to 90 days from randomisation and later undergo a follow‐up assessment at 180 days. During this assessment HRQoL is measured using the EQ‐5D‐5L questionnaire and EQ‐VAS by telephone interview, or, if not possible, by mail. Responses can be obtained from proxies (using the proxy‐participant view) if participants are unable to respond themselves.

The first participant in this study was enrolled on 9 March 2026, and enrolment will continue until 100 participants have been enrolled, which is expected to be by September 2026. Data collection will continue until data for 6 months of follow‐up is collected for all participants (Figure 1), i.e., 6 additional months after completion of the 180‐day INCEPT follow up (so for a total of 12 months from INCEPT first domain inclusion).

**FIGURE 1:**
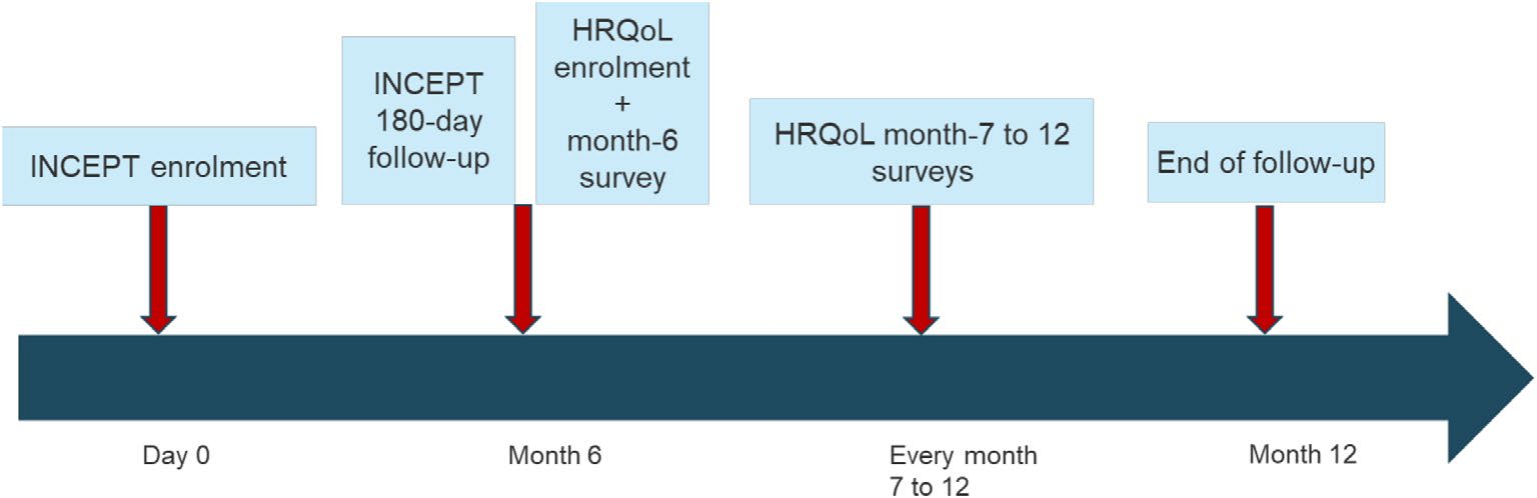
Timeline of the pilot study as embedded in INCEPT. INCEPT, Intensive Care Platform Trial [34]; HRQoL, health‐related quality of life.

### 2.2 Participants

We will seek to include all consecutive INCEPT participants enrolled at the coordinating Danish site (Copenhagen University Hospital—Rigshospitalet), the Aalborg site or the Hvidovre site, who are alive, give consent to data collection, and are undergoing the 180‐day telephone interview. We will exclude all patients who do not own a mobile device suitable for receiving links to the online survey via SMS or accessing the online survey. Also, all participants who are deemed, by the staff conducting the follow‐up interviews, incapable of personally responding to the EQ‐5D‐5L questionnaire via a web‐based form are excluded.

### 2.3 Data Collection and Measurements

Survey responses will be recorded in a bespoke REDCap database merged with data from the INCEPT electronic case report form (eCRF) for final analyses. To quantify HRQoL we will use the EQ‐5 Domain‐5‐Level (EQ‐5D‐5L) instrument with its visual analogue scale (EQ VAS) component designed for REDCap and made available by the EuroQoL group. EQ‐5D‐5L is a generic utility‐based tool that examines 5 domains: mobility, self‐care, usual activities, pain/discomfort, and anxiety/depression [35]. For each domain, the severity is chosen from a 5‐level Likert scale: none, slight, moderate, severe, and extreme problems. Profiles are collected as 5‐digit series of numbers, each representing the severity assigned to each domain by the participant in the order the domains are presented (e.g., 11111). Index values are used to summarise utility and calculated from EQ‐5D‐5L profiles using the Danish EQ‐5D‐5L value set [36], due to primarily Danish participants. They are anchored at 1.0 (perfect health), and 0 (a health state considered as bad as being dead), with values below 0 assigned to health statuses considered worse than death. EQ VAS score is the value assigned to perceived health on the day of the observation on a visual scale of 0 (worst) to 100 (best) health states [37].

We will send one text message monthly, starting from the day of enrolment in this study (or 180‐day INCEPT follow up) for 6 months, containing a link to the REDCap supported version of EQ‐5D‐5L + EQ VAS survey to be self‐completed by the recipient. We will send one SMS reminder after a week if the survey is not completed in that timeframe as a retention strategy (Figure 2). As part of the development of this SMS system, we engaged an ICU survivor to assess its accessibility and user experience, and subsequently incorporated revisions based on their feedback.

**FIGURE 2:**
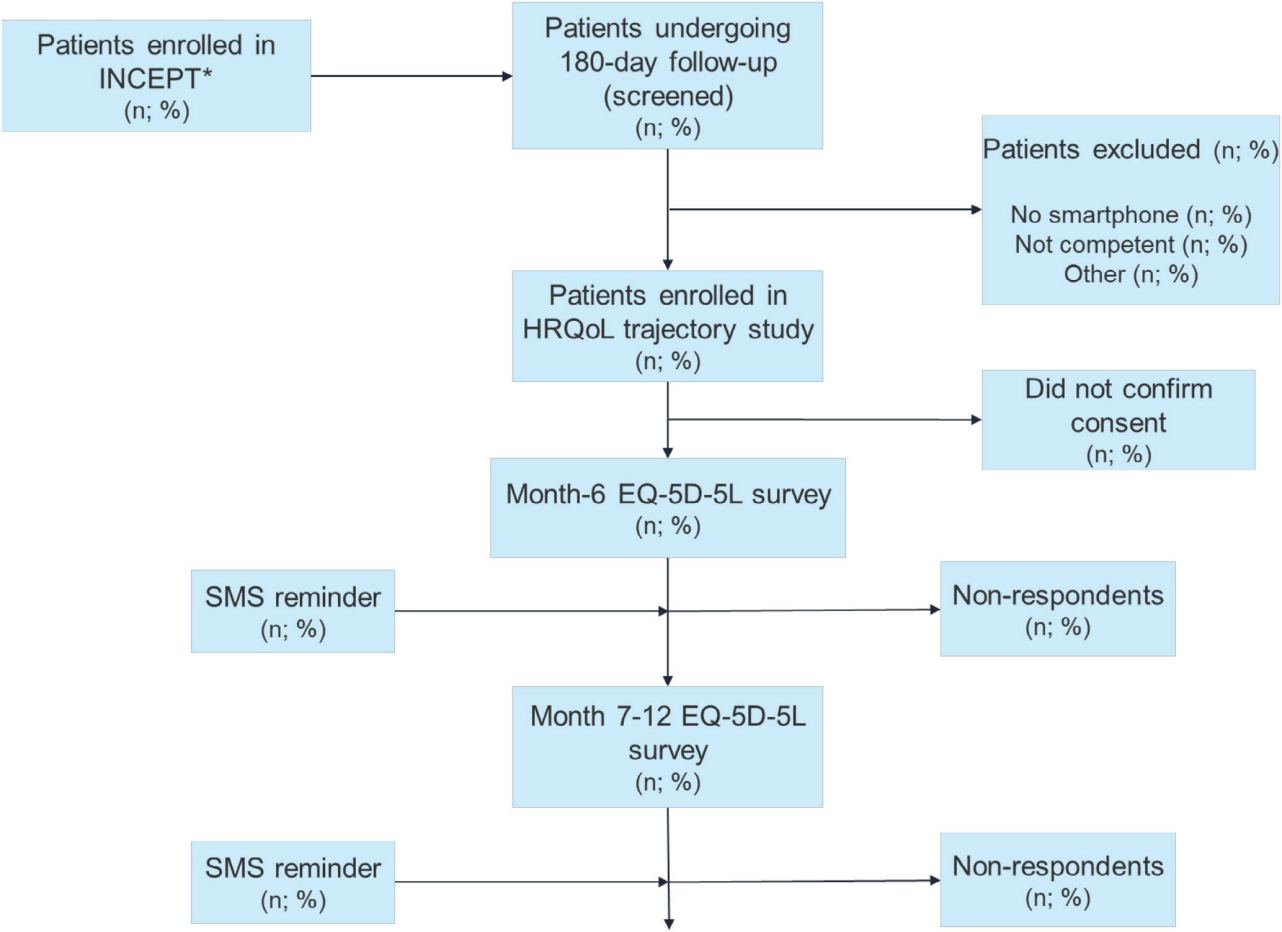
Patient flowchart. INCEPT, Intensive Care Platform Trial [34]; HRQoL, health‐related quality of life; EQ‐5D‐5L, EuroQol 5‐D 5‐Level [35]; SMS, short message service. *This includes all patients enrolled in INCEPT who would have reached the 180‐day follow‐up time point from March 9th, 2026, onward, irrespective of vital status or follow‐up completion, including deceased patients, patients who withdrew consent for continued data collection, and patients lost to follow‐up.

Data collected at the time of randomisation in INCEPT and at the time of inclusion in this study (i.e., at the time of 180‐day INCEPT follow‐up) will be as reported in Tables 1 and 2. We will register the main reason for declined consent to participate in this study (collected at 180‐day INCEPT telephone call), absence of suitable technological device (i.e., no smartphone or no internet access), inability to self‐complete the survey for those who are excluded.

**TABLE 1:** Baseline characteristics of patients enrolled.

Characteristic	Participants (*N*=)
Site of enrolment	
Rigshospitalet (*n*; %)	###
Aalborg (*n*; %)	###
Hvidovre (*n*; %)	###
INCEPT domains	
Albumin (*n*; %)	###
Thromboprophylaxis (*n*; %)	###
Both (*n*; %)	###
180‐day INCEPT telephone interview	
EQ‐5D‐5L Index values^a^ (median; IQR)	###
EQ VAS^a^ (median; IQR)	###
INCEPT baseline characteristics	
Age (median; IQR)	###
Female sex (*n*; %)	###
Weight kg (median; IQR)	###
Height m (median; IQR)	###
Use of invasive mechanical ventilation (*n*; %)	###
Use of vasopressors/inotropes (*n*; %)	###
Use of renal replacement therapy (RRT) (*n*; %)	###
Limitation of care (*n*; %)	###
Co‐existing conditions (*n*; %)	
Active haematological malignancy or metastatic cancer	###
History of ischemic heart disease or heart failure	###
Diabetes mellitus	###
Chronic pulmonary disease	###
Chronic liver disease	###
Known use of immunosuppressive therapy within the last 3 months	###
Previous organ transplantation	###
Chronic use of RRT	###
Treatment with antipsychotic at hospital admission	###
Acute surgery ≤ 7 days prior to randomisation (*n*; %)	###
SMS‐ICU^b^ (median; IQR)	###
Clinical Frailty Scale^c^ level (median; IQR)	###
Lowest systolic blood pressure in the 24 h preceding randomisation mmHg (median; IQR)	###
Highest plasma lactate in the 24 h prior to randomisation mmol/L (median; IQR)	###
Highest plasma creatine in the 24 h prior to randomisation μmol/L (median; IQR)	###

*Note:* It contains data collected at the time of inclusion in this study (site of enrolment, INCEPT domains, 180‐day telephone interview) and data extracted from INCEPT baseline characteristics collected at the time of first INCEPT screening. Results reported as counts; proportion for categorical data and median; interquartile range (IQR) for continuous data.

Abbreviations: CFS, Clinical Frailty Scale [38]; EQ VAS, EuroQol Visual Analogue Scale [35]; EQ‐5D‐5L, EuroQol 5‐D 5‐Level [35]; INCEPT, Intensive Care Platform Trial [34]; mmHg, millimetres of mercury; mmol/L, millimoles per Liter; RRT, renal replacement therapy; SMS‐ICU, Simplified Mortality Score for the Intensive care Unit [39]; μmol/L, micromoles per Liter.

^a^ Values ranging from 1.0 (perfect health, corresponding to a EQ‐5D‐5L profile of 11111) to −0.757 (worst possible value according to the Danish Value Set [36], corresponding to a EQ‐5D‐5L profile of 55555). EQ VAS score ranges from 0 (worst) to 100 (best) perceived health‐state.

^b^ Severity score ranging from 0 to 42 points, with higher scores indicating more severe illness and higher risk of death.

^c^ Investigator‐assessed clinical frailty, using version the Clinical Frailty Scale version 2 with 9 levels from 1 (very fit) to 9 (terminally ill).

**TABLE 2:** Description of HRQoL EQ‐5D‐5L baseline profiles, summarised per domain and level of problems.

	Mobility (*n*; %)	Self‐care (*n*; %)	Usual activities (*n*; %)	Pain/discomfort (*n*; %)	Anxiety/Depression (*n*; %)
Level 1 (No problems)	###	###	###	###	###
Level 2 (Slight problems)	###	###	###	###	###
Level 3 (Moderate problems)	###	###	###	###	###
Level 4 (Severe problems)	###	###	###	###	###
Level 5 (Extreme problem/unable to do)	###	###	###	###	###
Total	###	###	###	###	###

*Note:* Results reported as counts: proportions.

EuroQol 5‐D 5‐Level [35] is a generic utility‐based tool that examines 5 domains: mobility, self‐care, usual activities, pain/discomfort, and anxiety/depression. For each domain, the severity is chosen from a 5‐level Likert scale: none, slight, moderate, severe, and extreme problems. Profiles are collected as 5‐digit series of numbers, each representing the severity assigned to each domain by the participant in the order the domains are presented (e.g., 11111).

### 2.4 Outcomes

Feasibility will be assessed across the following domains:

1. Response rate, defined as proportion of participants completing the seven surveys (month 6, 7, 8, 9, 10, 11, 12 after randomisation^1^).
2. Enrolment rate, defined as proportion of enrolled patients among those screened (INCEPT participants who undergo INCEPT 180‐day follow‐up at the sites of this study within the period of enrolment to this study).
3. Time to completion, defined as response time from survey invitation to survey completion (for each survey).
4. Reminder‐dependency, defined as proportion of respondents who completed the survey after receival of SMS reminder (for each survey).
5. Attrition pattern categories, that is how loss to follow‐up occurred (complete data, monotone dropout, intermittent missing, no follow‐up), (Table 3).
6. Agreement, defined as comparability of interview and self‐report of data at month‐6 survey (or day‐180 from INCEPT enrolment).
7. Accessibility, defined as the proportion of patients screened with access to a suitable technological device.

**TABLE 3:** Exemplification of attrition patterns.

Participant	t6		t7		t8		t9		t10–12		
Pt 1	+		+		−	+	+		+		Complete data
Pt 2	+		−	+	+		−	−	−	−	Monotone dropout
Pt 3	−	+	−	−	+		−	+	−	+	Intermittent missing
Pt 4	−	−									No‐follow‐up/withdrawal

*Note:* For each survey timepoint, responses to initial SMS survey and 1‐week reminder are listed.

Complete data are defined as responses to all seven surveys (t6–t12). Monotone dropout is defined as a participant who completes one or more surveys and subsequently ceases responding to all remaining surveys. Intermittent missing data occur when a participant fails to respond at one or more timepoints but subsequently resumes. Patients classified as no follow‐up/withdrawn are those who received the initial survey but never provided a response.

Abbreviations: + completed; − not completed; Pt., patient; t6 month‐6 survey; t7 month‐7 survey; t8 month‐8 survey; etc.

A summary of reasons from eligible patients that are not enrolled will be provided.

Furthermore, we will investigate EQ‐5D‐5L area‐under‐the‐curve (AUC) for index values as well as VAS and their variability numerically (standard deviation, SD). We expect AUC to exhibit sufficient between‐subject variability to discriminate between patients with differing health trajectories, which would suggest its usefulness as a summary measure of HRQoL.

Finally, we will report EQ‐5D‐5L profiles as exploratory outcomes, changes over time, and EQ VAS descriptive statistics.

### 2.5 Study Size

The target sample size of 100 participants invited to the survey was chosen pragmatically to balance feasibility of recruitment within an appropriate timeframe with adequate statistical precision for key feasibility outcomes, consistent with recommendations for pilot and feasibility studies [32, 40]. For proportion‐based outcomes (response rate, enrolment rate), assuming a conservative expected proportion of 50%, a sample size of 100 participants provides approximately 10% absolute precision (i.e., from approximately 40%–60%) for a 95% confidence interval. For continuous outcomes (time to completion), no formal variance assumptions were made, and estimates will be considered exploratory. Overall, this sample size was considered sufficient to provide preliminary estimates of feasibility parameters while allowing for pragmatic recruitment within the study setting.

### 2.6 Statistical Methods

Feasibility results will be presented descriptively by counts (proportions) for categorical data and medians (interquartile ranges) for numerical data. We will not use predetermined thresholds for feasibility as this is an exploratory pilot study. Repeated measurements of feasibility outcomes will be displayed graphically by participant, without further statistical analysis at this stage.

Agreement between 180‐day telephone interview and month‐6 survey will be assessed at the group level by estimating the adjusted mean difference between modes using split‐plot analysis of variance (ANOVA) [41] and compared against predefined equivalence margins. As a minimally important difference (MID) specific for the ICU population is not available, we defined MID for EQ‐5D‐5L index value as 0.065 and MID for EQ VAS as 9.0 based on data from a recent systematic review of studies with original estimation of MIDs in a wide range of patient populations [42]. At the individual level, agreement will be evaluated using the intraclass correlation coefficient (ICC [3,1] [41]), derived from an ANOVA model including factors for mode and subject, and a two‐sided 95% CI will be provided.

Trajectories for HRQoL index values and VAS defined by linear interpolation will be used to estimate mean area‐under‐the‐curve (AUC) and its crude variance in the study population. HRQoL index values and VAS will be set at 0 for baseline (initial INCEPT randomisation).

HRQoL profiles over time will be summarised in a matrix table and Paretian Classification of Health Change (PCHC) [43] will be used to compare HRQoL at different time points. Changes will be classified as better (improvement in at least one domain), worse (deterioration in at least one domain), mixed changes (both improvement and deterioration in different domains), or no change. Missing data will be reported as counts with percentages, stratified by time point. Single imputation using linear interpolation will be applied for intermittent missing values, while last observation carried forward (LOCF) will be used for participants with monotone dropout. As death data are not available in this study, monotone dropout will include both participants who withdrew and those who may have died during follow‐up since these cannot be distinguished. LOCF will therefore be applied uniformly to this group, acknowledging that where dropout is attributable to death, this approach will tend to overestimate true HRQoL.

To quantify the potential impact of unobserved mortality on HRQoL estimates, two pre‐specified scenario‐based sensitivity analyses will be conducted in addition to the primary analysis:

1. No deaths assumed among dropouts (primary analysis)—LOCF is applied to all monotone dropouts, representing the most optimistic scenario, equivalent to the above‐mentioned primary analysis. This serves as the upper bound on HRQoL estimates.
2. All dropouts assumed dead—monotone dropouts are assigned death‐equivalent values (i.e., 0 for HRQoL index values) and worst‐possible values (i.e., 0 for VAS). This will serve as the lower bound on HRQoL estimates.
3. Probabilistic scenario—to explore intermediate assumptions, a range of plausible mortality fractions among dropouts (10%, 25%, 50%, 75%) will be examined. For each fraction, dropout participants will be randomly allocated to either the ‘dead’ group (assigned death‐equivalent values, or worst‐possible values for VAS) or the ‘alive’ group (assigned LOCF values), and AUC and trajectory estimates will be computed. This random allocation will be repeated across 1000 iterations for each assumed mortality fraction, generating a distribution of estimates that reflects uncertainty about the true proportion of deaths among dropouts. Results will be summarised as the mean estimate and 95% interval across iterations for each assumed fraction and presented graphically to illustrate how sensitive the primary conclusions are to assumptions about dropout composition.

Together, these scenarios define a plausible range for HRQoL trajectory and AUC estimates under different assumptions about unobserved mortality. For the purposes of this study, INCEPT domain and intervention arm allocation will be disregarded.

### 2.7 Ethics and Reporting

The study and data handling are approved by the Legal Department at Copenhagen University Hospital—Rigshospitalet, Copenhagen, Denmark. Consent from participants will be obtained during the 180‐day INCEPT follow‐up interview. They will orally consent to receiving the first survey and a reminder. If no response to the first survey and reminder is obtained, consent will be considered withdrawn.

## 3 Discussion

HRQoL is an important outcome for ICU survivors. Efforts to optimise data collection methods, maximise retention, and reduce missingness are essential, while ensuring that the associated human and financial resources requirements remain acceptable. We propose a follow‐up method that supports these goals by enabling easily repeatable measurements, promoting retention, and reducing attrition through a minimally invasive approach, and allowing patients to respond at their convenience. To our knowledge, this is the first study to explore the use of ePRO methods for this patient population.

Given the exploratory nature of this pilot study, we did not predefine formal feasibility thresholds. Instead, our findings are intended to inform a broader assessment of feasibility and guide future study design. Several uncertainties remain, including the acceptability and accessibility of ePRO methods in a population with substantial post‐ICU morbidity, and whether HRQoL trajectories represent a viable approach to capture both improved survival and patterns of recovery. Further work is warranted once the broader feasibility has been elucidated.

In addition, we aimed to explore a candidate approach to HRQoL analysis that may provide a more comprehensive and patient‐centered assessment of post‐ICU outcomes and possibly better capture the effects of survival‐improving interventions in this setting. Given the marked heterogeneity of HRQoL over time, the use of longitudinal measurements and their integration as an AUC avoids the limitations of single early or late assessments, instead capturing the entire recovery trajectory, including fluctuations. As critical illness is associated with long‐lasting, multi‐domain consequences, the AUC approach enables aggregation of time spent in impaired health states and therefore better reflects prolonged disability, recurrent complications, and delayed recovery. Adopting this methodology, conceptually similar to quality‐adjusted life years (QALYs) but applying it outside of the scope of economic evaluations, may enhance the implementation, interpretability, and clinical relevance of HRQoL outcomes in intensive care RCTs.

### 3.1 Strengths and Limitations

This study will have several strengths. First, data analysis will be conducted according to this pre‐specified protocol, although patient enrolment has begun prior to its completion and publication. Second, we have designed a solid patient‐centred setup to maximise convenience, with short completion times and flexibility for participants to respond at their preferred time. Third, during the development phase, stakeholder involvement was implemented to ensure accessibility and usability. Fourth, we will compare ePRO responses with standard PRO collection via telephone interviews to assess agreement to further qualify our findings. Fifth, participants are enrolled in three different sites with different levels of specialisation (a specialised tertiary referral hospital, a specialised university hospital and a large regional acute hospital), which improves the external validity of the results.

The study also has certain limitations. First, as a pilot study, obstacles may arise that we had not anticipated or planned for. Although this may result in fewer data points that might be desired, it would enable us to improve the procedure before a large‐scale, genuine feasibility study is undertaken. Second, we cannot collect pre‐ICU HRQoL data, which precludes adjustment for baseline status. Since ICU admission in Denmark generally, and inclusion in INCEPT particularly, follows critical illness, setting a baseline HRQoL equivalent to death seems a reasonable assumption. Third, to maintain a simple study design we did not implement any additional retention strategies (e.g., combining SMS, email, and telephone follow‐up), which might limit accessibility to patients who do not own a smartphone or internet connection. This aspect, together with other factors potentially influencing participation in the study, could result in the inclusion of a selected survivor population. Fourth, due to data protection regulation, we will not have access to mortality data during the study period. Consequently, all patients will be classified as non‐respondents (monotone dropout) regardless of their survival status. The pre‐planned sensitivity analyses will help mitigate the introduced bias by allowing to evaluate the robustness of the primary findings without access to formal survival data. Fifth, disregarding INCEPT domain and intervention allocation, prevents us from capturing their potential effects on HRQoL trajectories.

### 3.2 Conclusions

In conclusion, this pilot study will be the first of its kind to assess the feasibility of collecting ePRO HRQoL data via repeated sampling through text message in ICU survivors. It will also provide preliminary evidence on the feasibility of quantifying long‐term HRQoL trajectories, potentially offering a more comprehensive representation of the dynamic nature of HRQoL over time in a single summary measure.

## Author Contributions

**Elisa Zoe Battistelli**, **Benjamin Skov Kaas‐Hansen**, **Maj‐Brit Nørregaard Kjær:** conceptualisation. **Elisa Zoe Battistelli**, **Benjamin Skov Kaas‐Hansen:** formal analysis. **Elisa Zoe Battistelli**, **Benjamin Skov Kaas‐Hansen**, **Maj‐Brit Nørregaard Kjær:** methodology. **Elisa Zoe Battistelli:** writing – original draft. **Anders Granholm**, **Ronni Thermann Reitz Plovsing**, **Bodil Steen Rasmussen**, **Maj‐Brit Nørregaard Kjær:** writing – review and editing. **Anders Perner**, **Benjamin Skov Kaas‐Hansen**, **Maj‐Brit Nørregaard Kjær:** supervision.

## Funding

The INCEPT research program is funded by the Novo Nordisk Foundation and Sygeforsikringen ‘danmark’, with additional support by Grosserer Jakob Ehrenreich og Hustru Grete Ehrenreichs Fond, Dagmar Marshalls fond, and Savværksejer Jeppe Juhl og hustru Ovita Juhls Mindelegat. The INCEPT‐Albumin domain is partially funded by Danmarks Frie Forskningsfond. The INCEPT‐Thromboprophylaxis domain is additionally funded by the Netherlands Thrombosis Foundation (2025_04).

## Conflicts of Interest

The Department of Intensive Care, Rigshospitalet receives funding from the Novo Nordisk Foundation, Sygeforsikringen ‘danmark’, and Becketts Foundation for other projects. The other authors declare no conflicts of interest.

## Supporting information


**Table S1:** STROBE statement checklist for cohort studies, modified from STROBE Initiative website http://www.strobe‐statement.org. Items reported per section number.
**Table S2:** CONSORT checklist of information to include when reporting a pilot trial, modified from Eldridge SM, Chan CL, Campbell MJ, et al. CONSORT 2010 statement: extension to randomised pilot and feasibility trials. BMJ. Published online October 24, 2016: i5239. doi:10.1136/bmj.i5239. Items reported per section number.

## Data Availability

The data that support the findings of this study are available from the corresponding author upon reasonable request.

## Endnotes

^1^
INCEPT 180‐day follow‐up may be conducted within ±14 days of day 180 after randomisation. The month 1 survey is sent on the same day, and each subsequent survey is sent 30 days after completion of the previous survey.

## References

[1] D. M. Needham , J. Davidson , H. Cohen , et al., “Improving Long‐Term Outcomes After Discharge From Intensive Care Unit: Report From a stakeholders Conference,” Critical Care Medicine 40, no. 2 (2012): 502–509, 10.1097/CCM.0b013e318232da75. 21946660

[2] M. Calvert , D. Kyte , R. Mercieca‐Bebber , et al., “Guidelines for Inclusion of Patient‐Reported Outcomes in Clinical Trial Protocols: The SPIRIT‐PRO Extension,” JAMA 319, no. 5 (2018): 483–494, 10.1001/jama.2017.21903. 29411037

[3] M. Calvert , M. King , R. Mercieca‐Bebber , et al., “SPIRIT‐PRO Extension Explanation and Elaboration: Guidelines for Inclusion of Patient‐Reported Outcomes in Protocols of Clinical Trials,” BMJ Open 11, no. 6 (2021): e045105, 10.1136/bmjopen-2020-045105. PMC8246371 34193486

[4] M.‐B. N. Kjær , C. R. L. Bruun , A. Granholm , et al., “A Core Outcome Set for Adult General ICU Patients,” Critical Care Medicine 53, no. 3 (2025): e575–e589, 10.1097/CCM.0000000000006556. 40036020

[5] L. Rose , L. Burry , M. Agar , et al., “A Core Outcome Set for Research Evaluating Interventions to Prevent and/or Treat Delirium in Critically Ill Adults: An International Consensus Study (Del‐COrS),” Critical Care Medicine 49, no. 9 (2021): 1535–1546, 10.1097/CCM.0000000000005028. 33870914

[6] D. M. Needham , K. A. Sepulveda , V. D. Dinglas , et al., “Core Outcome Measures for Clinical Research in Acute Respiratory Failure Survivors. An International Modified Delphi Consensus Study,” American Journal of Respiratory and Critical Care Medicine 196, no. 9 (2017): 1122–1130, 10.1164/rccm.201702-0372OC. PMC5694837 28537429

[7] A. Granholm , C. T. Anthon , M. B. N. Kjær , et al., “Patient‐Important Outcomes Other Than Mortality in Contemporary ICU Trials: A Scoping Review,” Critical Care Medicine 50, no. 10 (2022): e759–e771, 10.1097/CCM.0000000000005637. 35894598

[8] J. G. M. Hofhuis , H. F. Van Stel , A. J. P. Schrijvers , J. H. Rommes , and P. E. Spronk , “ICU Survivors Show no Decline in Health‐Related Quality of Life After 5 Years,” Intensive Care Medicine 41, no. 3 (2015): 495–504, 10.1007/s00134-015-3669-5. 25672277

[9] B. H. Cuthbertson , S. Roughton , D. Jenkinson , G. MacLennan , and L. Vale , “Quality of Life in the Five Years After Intensive Care: A Cohort Study,” Critical Care 14, no. 1 (2010): R6, 10.1186/cc8848. PMC2875518 20089197

[10] T. J. Iwashyna , “Trajectories of Recovery and Dysfunction After Acute Illness, With Implications for Clinical Trial Design,” American Journal of Respiratory and Critical Care Medicine 186, no. 4 (2012): 302–304, 10.1164/rccm.201206-1138ED. PMC3443808 22896591

[11] O. Pallanch , A. Ortalda , P. Pelosi , et al., “Effects on Health‐Related Quality of Life of Interventions Affecting Survival in Critically Ill Patients: A Systematic Review,” Critical Care 26, no. 1 (2022): 126, 10.1186/s13054-022-03993-3. PMC9075706 35524315

[12] E. Colantuoni , D. O. Scharfstein , C. Wang , et al., “Statistical Methods to Compare Functional Outcomes in Randomized Controlled Trials With High Mortality,” BMJ 360 (2018): j5748, 10.1136/bmj.j5748. PMC5751848 29298779

[13] M. Herdman , C. Gudex , A. Lloyd , et al., “Development and Preliminary Testing of the New Five‐Level Version of EQ‐5D (EQ‐5D‐5L),” Quality of Life Research 20, no. 10 (2011): 1727–1736, 10.1007/s11136-011-9903-x. PMC3220807 21479777

[14] E. Stolk , K. Ludwig , K. Rand , B. Van Hout , and J. M. Ramos‐Goñi , “Overview, Update, and Lessons Learned From the International EQ‐5D‐5L Valuation Work: Version 2 of the EQ‐5D‐5L Valuation Protocol,” Value in Health 22, no. 1 (2019): 23–30, 10.1016/j.jval.2018.05.010. 30661630

[15] N. Devlin , D. Parkin , and B. Janssen , Methods for Analysing and Reporting EQ‐5D Data (Springer International Publishing, 2020). 33347096

[16] J. Yan , B. Humphries , R. Xie , et al., “Statistical Methods for Analyzing EQ‐5D in Randomized Clinical Trials: A Systematic Literature Review,” Value in Health 28, no. 7 (2025): 1126–1135, 10.1016/j.jval.2025.02.001. 39954858

[17] L. J. Cheng , T. Pan , L. A. Chen , et al., “The Ceiling Effects of EQ‐5D‐3L and 5L in General Population Health Surveys: A Systematic Review and Meta‐Analysis,” Value in Health 27, no. 7 (2024): 986–997, 10.1016/j.jval.2024.02.018. 38467187

[18] M. Powney , P. Williamson , J. Kirkham , and R. Kolamunnage‐Dona , “A Review of the Handling of Missing Longitudinal Outcome Data in Clinical Trials,” Trials 15, no. 1 (2014): 237, 10.1186/1745-6215-15-237. PMC4087243 24947664

[19] M. N. Kjær , M. B. Madsen , N. Meier , et al., “Non‐Response for Health‐Related Quality of Life Outcomes in ICU Patients: A Systematic Review of the Reporting in Randomised Trials,” Acta Anaesthesiologica Scandinavica 67, no. 7 (2023): 842–852, 10.1111/aas.14250. 37078459

[20] M. N. Kjær , C. B. Mortensen , P. B. Hjortrup , S. L. Rygård , I. Andersen , and A. Perner , “Factors Associated With Non‐Response at Health‐Related Quality of Life Follow‐Up in a Septic Shock Trial,” Acta Anaesthesiologica Scandinavica 62, no. 3 (2018): 357–366, 10.1111/aas.13056. 29282713

[21] E. Colantuoni , X. Li , M. D. Hashem , T. D. Girard , D. O. Scharfstein , and D. M. Needham , “A Structured Methodology Review Showed Analyses of Functional Outcomes Are Frequently Limited to ‘Survivors Only’ in Trials Enrolling Patients at High Risk of Death,” Journal of Clinical Epidemiology 137 (2021): 126–132, 10.1016/j.jclinepi.2021.03.027. PMC8464482 33838275

[22] N. Hubel , D. Krepper , L. van der Weijst , et al., “Assessing the Assessment: A Scoping Review of the Mode of Patient‐Reported Outcome Assessment in Solid Cancer Clinical Trials,” Quality of Life Research 35, no. 4 (2026): 80, 10.1007/s11136-026-04179-y. PMC12950655 41764668

[23] S. J. Coons , C. J. Gwaltney , R. D. Hays , et al., “Recommendations on Evidence Needed to Support Measurement Equivalence Between Electronic and Paper‐Based Patient‐Reported Outcome (PRO) Measures: ISPOR ePRO Good Research Practices Task Force Report,” Value in Health 12, no. 4 (2009): 419–429, 10.1111/j.1524-4733.2008.00470.x. 19900250

[24] R. Mercieca‐Bebber , M. J. Palmer , M. Brundage , M. Calvert , M. R. Stockler , and M. T. King , “Design, Implementation and Reporting Strategies to Reduce the Instance and Impact of Missing Patient‐Reported Outcome (PRO) Data: A Systematic Review,” BMJ Open 6, no. 6 (2016): e010938, 10.1136/bmjopen-2015-010938. PMC4916640 27311907

[25] S. J. Coons , S. Eremenco , J. J. Lundy , P. O'Donohoe , H. O'Gorman , and W. Malizia , “Capturing Patient‐Reported Outcome (PRO) Data Electronically: The Past, Present, and Promise of ePRO Measurement in Clinical Trials,” Patient ‐ Patient‐Centered Outcomes Research 8, no. 4 (2015): 301–309, 10.1007/s40271-014-0090-z. PMC4529477 25300613

[26] C. J. Gwaltney , A. L. Shields , and S. Shiffman , “Equivalence of Electronic and Paper‐and‐Pencil Administration of Patient‐Reported Outcome Measures: A Meta‐Analytic Review,” Value in Health 11, no. 2 (2008): 322–333, 10.1111/j.1524-4733.2007.00231.x. 18380645

[27] W. Muehlhausen , H. Doll , N. Quadri , et al., “Equivalence of Electronic and Paper Administration of Patient‐Reported Outcome Measures: A Systematic Review and Meta‐Analysis of Studies Conducted Between 2007 and 2013,” Health and Quality of Life Outcomes 13, no. 1 (2015): 167, 10.1186/s12955-015-0362-x. PMC4597451 26446159

[28] F. D. Mowlem , C. A. Elash , K. M. Dumais , et al., “Best Practices for the Electronic Implementation and Migration of Patient‐Reported Outcome Measures,” Value in Health 27, no. 1 (2024): 79–94, 10.1016/j.jval.2023.10.007. 37879401

[29] K. Garner and B. Byrom , “Attitudes of Older People/Seniors to Completion of Electronic Patient‐Reported Outcome Measures and Use of Mobile Applications in Clinical Trials: Results of a Qualitative Research Study,” Journal of Comparative Effectiveness Research 9, no. 4 (2020): 307–315, 10.2217/cer-2019-0155. 32048516

[30] J. J. Ly , M. Crescioni , S. Eremenco , et al., “Training on the Use of Technology to Collect Patient‐Reported Outcome Data Electronically in Clinical Trials: Best Practice Recommendations From the ePRO Consortium,” Therapeutic Innovation & Regulatory Science 53, no. 4 (2019): 431–440, 10.1177/2168479018796206. 30221997

[31] E. von Elm , D. G. Altman , M. Egger , S. J. Pocock , P. C. Gøtzsche , and J. P. Vandenbroucke . “STROBE Initiative. Strengthening the Reporting of Observational Studies in Epidemiology (STROBE) Statement: Guidelines for Reporting Observational Studies,” BMJ 335, no. 7624 (2007): 806–808, 10.1136/bmj.39335.541782.AD. PMID: 17947786; PMCID: PMC2034723. PMC2034723 17947786

[32] S. M. Eldridge , C. L. Chan , M. J. Campbell , et al., “CONSORT 2010 Statement: Extension to Randomised Pilot and Feasibility Trials,” BMJ 355 (2016): i5239, 10.1136/bmj.i5239. PMC5076380 27777223

[33] S. Treweek , S. Bevan , P. Bower , et al., “Trial Forge Guidance 1: What Is a Study Within A Trial (SWAT)?,” Trials 19, no. 1 (2018): 139, 10.1186/s13063-018-2535-5. PMC5824570 29475444

[34] A. Granholm , M. H. Møller , B. S. Kaas‐Hansen , et al., “INCEPT: The Intensive Care Platform Trial—Design and Protocol,” Acta Anaesthesiologica Scandinavica 69, no. 4 (2025): e70023, 10.1111/aas.70023. PMC11907384 40084471

[35] N. Devlin , S. Pickard , and J. Busschbach , “The Development of the EQ‐5D‐5L and Its Value Sets,” in Value Sets for EQ‐5D‐5L, ed. N. Devlin , B. Roudijk , and K. Ludwig (Springer International Publishing, 2022), 1–12. 36810043

[36] C. E. Jensen , S. S. Sørensen , C. Gudex , M. B. Jensen , K. M. Pedersen , and L. H. Ehlers , “The Danish EQ‐5D‐5L Value Set: A Hybrid Model Using cTTO and DCE Data,” Applied Health Economics and Health Policy 19, no. 4 (2021): 579–591, 10.1007/s40258-021-00639-3. PMC8270796 33527304

[37] EuroQol Research Foundation , “EQ‐5D‐5L User Guide,” 2025, https://euroqol.org/information‐and‐support/documentation/user‐guides.

[38] K. Rockwood and O. Theou , “Using the Clinical Frailty Scale in Allocating Scarce Health Care Resources,” Canadian Geriatrics Journal 23, no. 3 (2020): 254–259, 10.5770/cgj.23.463. PMC7458601 32904824

[39] A. Granholm , A. Perner , M. Krag , et al., “Development and Internal Validation of the Simplified Mortality Score for the Intensive Care Unit (SMS ‐ ICU),” Acta Anaesthesiologica Scandinavica 62, no. 3 (2018): 336–346, 10.1111/aas.13048. 29210058

[40] R. Montgomery , “Sample Size Justification in Feasibility Studies: Moving Beyond Published Guidance,” Pilot and Feasibility Studies 11, no. 1 (2025): 88, 10.1186/s40814-025-01675-9. PMC12186404 40551266

[41] J. J. Lundy , S. J. Coons , E. Flood , and M. J. Patel , “Agreement Among Paper and Electronic Modes of the EQ‐5D‐5L,” Patient‐Patient‐Centered Outcomes Research 13, no. 4 (2020): 435–443, 10.1007/s40271-020-00419-6. PMC7340667 32346816

[42] F. Al Sayah , X. Jin , H. Short , N. S. McClure , A. Ohinmaa , and J. A. Johnson , “A Systematic Literature Review of Important and Meaningful Differences in the EQ‐5D Index and Visual Analog Scale Scores,” Value in Health 28, no. 3 (2025): 470–476, 10.1016/j.jval.2024.11.006. 39694263

[43] N. J. Devlin , D. Parkin , and J. Browne , Using the EQ‐5D as a Performance Measurement Tool in the NHS (City University London, 2009).

