# Supplementary material for: Electronic Patient‐Reported Health‐Related Quality of Life Trajectories in ICU Survivors: A Multicentre Pilot Study‐Within‐a‐Trial Protocol
Source: Acta Anaesthesiol Scand. 2026 Sep 29;70(10):e70342. doi: 10.1111/aas.70342 (PMC13623536; doi:10.1111/aas.70342)
Supplement: Supplementary file 1 [file AAS-70-0-s001.pdf]

Electronic patient-reported health-related quality of life trajectories in ICU survivors: a multicentre pilot study-within-a-trial protocol

Authors

Elisa Zoe Battistelli[1,2], Benjamin Skov Kaas-Hansen[1], Anders Granholm[1,3], Ronni Thermann Reitz Plovsing[4,5], Bodil Steen Rasmussen[6,7], Anders Perner[1,5], Maj-Brit Nørregaard Kjaer[1]

[1]Department of Intensive Care, Copenhagen University Hospital - Rigshospitalet, Copenhagen, Denmark

[2]Università degli Studi Milano-Bicocca, Milano, Italy

[3]Section of Biostatistics, Department of Public Health, University of Copenhagen, Copenhagen, Denmark

[4]Department of Anaesthesiology and Intensive Care, Copenhagen University Hospital – Amager and Hvidovre, Hvidovre, Denmark

[5]Department of Clinical Medicine, University of Copenhagen, Copenhagen, Denmark

[6] Department of Anaesthesiology and Intensive Care, Aalborg University Hospital, Aalborg University, Aalborg, Denmark

[7] Department of Clinical Medicine, Aalborg University, Aalborg, Denmark

CORRESPONDENCE
Elisa Zoe Battistelli

Department of Intensive Care 4131, Copenhagen University Hospital—Rigshospitalet, 2100 Copenhagen, Denmark. Università Degli Studi Milano – Bicocca, 20126, Milano, Italy

Email: elisa.zoe.battistelli@regionh.dk

SUPPLEMENTS

Table S1

	Item No	Recommendation	Section No
**Title and abstract**	1	(*a*) Indicate the study's design with a commonly used term in the title or the abstract<br><br>(*b*) Provide in the abstract an informative and balanced summary of what was done and what was found	TITLE,<br>ABSTRACT
**Introduction**			
Background/rationale	2	Explain the scientific background and rationale for the investigation being reported	1.1
Objectives	3	State specific objectives, including any prespecified hypotheses	1.2
**Methods**			
Study design	4	Present key elements of study design early in the paper	2.1
Setting	5	Describe the setting, locations, and relevant dates, including periods of recruitment, exposure, follow-up, and data collection	2.1<br>2.3
Participants	6	(a) Give the eligibility criteria, and the sources and methods of selection of participants. Describe methods of follow-up<br><br>(b) For matched studies, give matching criteria and number of exposed and unexposed	2.2
Variables	7	Clearly define all outcomes, exposures, predictors, potential confounders, and effect modifiers. Give diagnostic criteria, if applicable	2.4
Data sources/ measurement	8*	For each variable of interest, give sources of data and details of methods of assessment (measurement). Describe comparability of assessment methods if there is more than one group	2.3
Bias	9	Describe any efforts to address potential sources of bias	2.6
Study size	10	Explain how the study size was arrived at	2.5
Quantitative variables	11	Explain how quantitative variables were handled in the analyses. If applicable, describe which groupings were chosen and why	2.4
Statistical methods	12	(a) Describe all statistical methods, including those used to control for confounding<br><br>(b) Describe any methods used to examine subgroups and interactions	2.6

		(c) Explain how missing data were addressed<br>(d) If applicable, explain how loss to follow-up was addressed<br>(e) Describe any sensitivity analyses	
**Results**			N/a
Participants	13*	(a) Report numbers of individuals at each stage of study—eg numbers potentially eligible, examined for eligibility, confirmed eligible, included in the study, completing follow-up, and analysed<br>(b) Give reasons for non-participation at each stage<br>(c) Consider use of a flow diagram	
Descriptive data	14*	(a) Give characteristics of study participants (eg demographic, clinical, social) and information on exposures and potential confounders<br>(b) Indicate number of participants with missing data for each variable of interest<br>(c) Summarise follow-up time (eg, average and total amount)	
Outcome data	15*	Report numbers of outcome events or summary measures over time	
Main results	16	(a) Give unadjusted estimates and, if applicable, confounder-adjusted estimates and their precision (eg, 95% confidence interval). Make clear which confounders were adjusted for and why they were included	
		(b) Report category boundaries when continuous variables were categorized	
		(c) If relevant, consider translating estimates of relative risk into absolute risk for a meaningful time period	
Other analyses	17	Report other analyses done—eg analyses of subgroups and interactions, and sensitivity analyses	
**Discussion**			
Key results	18	Summarise key results with reference to study objectives	N/a
Limitations	19	Discuss limitations of the study, taking into account sources of potential bias or imprecision. Discuss both direction and magnitude of any potential bias	3.1
Interpretation	20	Give a cautious overall interpretation of results considering objectives, limitations, multiplicity of analyses, results from similar studies, and other relevant evidence	3
Generalisability	21	Discuss the generalisability (external validity) of the study results	3
**Other information**			

Funding	22	Give the source of funding and the role of the funders for the present study and, if applicable, for the original study on which the present article is based	FUNDING

N/a, not applicable

Table S2

Section/topic and item No	Standard checklist item	Extension for pilot trials	Section No where item is reported
**Title and abstract**			
1a	Identification as a randomised trial in the title	Identification as a pilot or feasibility randomised trial in the title	TITLE
1b	Structured summary of trial design, methods, results, and conclusions (for specific guidance see CONSORT for abstracts)	Structured summary of pilot trial design, methods, results, and conclusions (for specific guidance see CONSORT abstract extension for pilot trials)	ABSTRACT
**Introduction**			
Background and objectives:			
2a	Scientific background and explanation of rationale	Scientific background and explanation of rationale for future definitive trial, and reasons for randomised pilot trial	1.1
2b	Specific objectives or hypotheses	Specific objectives or research questions for pilot trial	1.2
**Methods**			
Trial design:			
3a	Description of trial design (such as parallel, factorial) including allocation ratio	Description of pilot trial design (such as parallel, factorial) including allocation ratio	2.1
3b	Important changes to methods after trial commencement (such as eligibility criteria), with reasons	Important changes to methods after pilot trial commencement (such as eligibility criteria), with reasons	N/a
Participants:			
4a	Eligibility criteria for participants		2.2
4b	Settings and locations where the data were collected		2.1
4c		How participants were identified and consented	2.2<br>2.7
Interventions:			
5	The interventions for each group with sufficient details to allow replication, including how and		2.3

	when they were actually administered		
Outcomes:			
6a	Completely defined prespecified primary and secondary outcome measures, including how and when they were assessed	Completely defined prespecified assessments or measurements to address each pilot trial objective specified in 2b, including how and when they were assessed	2.4
6b	Any changes to trial outcomes after the trial commenced, with reasons	Any changes to pilot trial assessments or measurements after the pilot trial commenced, with reasons	N/a
6c		If applicable, prespecified criteria used to judge whether, or how, to proceed with future definitive trial	N/a
Sample size:			
7a	How sample size was determined	Rationale for numbers in the pilot trial	2.5
7b	When applicable, explanation of any interim analyses and stopping guidelines		N/a
Randomisation:			
Sequence generation:			
8a	Method used to generate the random allocation sequence		N/a
8b	Type of randomisation; details of any restriction (such as blocking and block size)	Type of randomisation(s); details of any restriction (such as blocking and block size)	N/a
Allocation concealment mechanism:			
9	Mechanism used to implement the random allocation sequence (such as sequentially numbered containers), describing any steps taken to conceal the sequence until interventions were assigned		N/a
Implementation:			
10	Who generated the random allocation sequence, enrolled participants, and assigned participants to interventions		N/a
Blinding:			
11a	If done, who was blinded after assignment to interventions (eg, participants, care providers, those assessing outcomes) and how		N/a
11b	If relevant, description of the similarity of interventions		N/a
Analytical methods:			
12a	Statistical methods used to compare groups for primary and secondary outcomes	Methods used to address each pilot trial objective whether qualitative or quantitative	2.6
12b	Methods for additional analyses, such as subgroup analyses and adjusted analyses	Not applicable	N/a
**Results**			
Participant flow (a diagram is strongly recommended):			
13a	For each group, the numbers of participants who were randomly assigned, received intended treatment, and were analysed for the primary outcome	For each group, the numbers of participants who were approached and/or assessed for eligibility, randomly assigned, received intended treatment, and were assessed for each objective	N/a

13b	For each group, losses and exclusions after randomisation, together with reasons		N/a
Recruitment:			
14a	Dates defining the periods of recruitment and follow-up		2.1
14b	Why the trial ended or was stopped	Why the pilot trial ended or was stopped	N/a
Baseline data:			
15	A table showing baseline demographic and clinical characteristics for each group		TABLE 1,2
Numbers analysed:			
16	For each group, number of participants (denominator) included in each analysis and whether the analysis was by original assigned groups	For each objective, number of participants (denominator) included in each analysis. If relevant, these numbers should be by randomised group	N/a
Outcomes and estimation:			
17a	For each primary and secondary outcome, results for each group, and the estimated effect size and its precision (such as 95% confidence interval)	For each objective, results including expressions of uncertainty (such as 95% confidence interval) for any estimates. If relevant, these results should be by randomised group	N/a
17b	For binary outcomes, presentation of both absolute and relative effect sizes is recommended	Not applicable	N/a
Ancillary analyses:			
18	Results of any other analyses performed, including subgroup analyses and adjusted analyses, distinguishing prespecified from exploratory	Results of any other analyses performed that could be used to inform the future definitive trial	N/a
Harms:			
19	All important harms or unintended effects in each group (for specific guidance see CONSORT for harms)		N/a
19a		If relevant, other important unintended consequences	N/a
**Discussion**			
Limitations:			
20	Trial limitations, addressing sources of potential bias, imprecision, and, if relevant, multiplicity of analyses	Pilot trial limitations, addressing sources of potential bias and remaining uncertainty about feasibility	3.1
Generalisability:			
21	Generalisability (external validity, applicability) of the trial findings	Generalisability (applicability) of pilot trial methods and findings to future definitive trial and other studies	3
Interpretation:			
22	Interpretation consistent with results, balancing benefits and harms, and considering other relevant evidence	Interpretation consistent with pilot trial objectives and findings, balancing potential benefits and harms, and considering other relevant evidence	N/a

22a		Implications for progression from pilot to future definitive trial, including any proposed amendments	
**Other information**			
Registration:			
23	Registration number and name of trial registry	Registration number for pilot trial and name of trial registry	N/a
Protocol:			
24	Where the full trial protocol can be accessed, if available	Where the pilot trial protocol can be accessed, if available	N/a
Funding:			
25	Sources of funding and other support (such as supply of drugs), role of funders		FUNDING
26		Ethical approval or approval by research review committee, confirmed with reference number	2.7

N/a, not applicable
